# Landscape of Immune-Related Markers and Potential Therapeutic Targets in Soft Tissue Sarcoma

**DOI:** 10.3390/cancers13205249

**Published:** 2021-10-19

**Authors:** Jason Roszik, Lisa Maria Mustachio, John A. Livingston, Roman Groisberg, Roberto Carmagnani Pestana, Vivek Subbiah, Anthony P. Conley

**Affiliations:** 1 Department of Genomic Medicine, Division of Cancer Medicine, The University of Texas MD Anderson Cancer Center, Houston, TX 77030, USA; 2 Department of Melanoma Medical Oncology, Division of Cancer Medicine, The University of Texas MD Anderson Cancer Center, Houston, TX 77030, USA; 3 Department of Epigenetics and Molecular Carcinogenesis, The University of Texas MD Anderson Cancer Center, Houston, TX 77030, USA; Lmustachio@mdanderson.org; 4 Center for Cancer Epigenetics, The University of Texas MD Anderson Cancer Center, Houston, TX 77030, USA; 5 Department of Sarcoma Medical Oncology, Division of Cancer Medicine, The University of Texas MD Anderson Cancer Center, Houston, TX 77030, USA; JALivingston@mdanderson.org; 6 Department of Sarcoma/Melanoma Medical Oncology, Rutgers Cancer Institute of New Jersey, 195 Little Albany St., New Brunswick, NJ 08903, USA; roman.groisberg@rutgers.edu; 7 Department of Investigational Cancer Therapeutics, The University of Texas MD Anderson Cancer Center, 1515 Holcombe Blvd., Houston, TX 77030, USA; robertocpestana@gmail.com (R.C.P.); vsubbiah@mdanderson.org (V.S.)

**Keywords:** soft tissue sarcoma, immunotherapy, immune checkpoint, next-generation sequencing, mutational burden, TCGA

## Abstract

**Simple Summary:**

Despite being a group of rare diseases of mesenchymal origin, soft tissue sarcomas are heterogenous and display varying clinical behavior, and depending on the subtype, intermediate- and high-grade sarcomas have significant metastatic potential, making it difficult to establish a standardized therapy. Our work, as well as studies by others, emphasizes the high potential of immunotherapy for the treatment of sarcoma. The aim of this study was to determine whether specific genomic alterations, as well as the expression of infiltrating cytotoxic and suppressive cell type markers identified by next-generation sequencing (NGS), warrant further consideration of immunotherapy agents for treating certain soft tissue sarcoma subtypes. Altogether, our data provide a better understanding of the immune composition of different sarcoma subtypes to better identify novel therapy targets.

**Abstract:**

Soft tissue sarcomas, depending on the subtype and grade, frequently recur and become metastatic after localized treatment. There is now great interest in applying immunotherapy to sarcomas to immuno-profile the different subtypes and immune monitor for prognosis. Our group previously showed that key immunotherapy target genes are present in sarcomas. Here, we extend our findings by demonstrating that sarcomas with a relatively high mutational load are likely to be more sensitive to immunotherapy compared to sarcomas with a lower mutation load. We also show that sarcomas with a higher mutation load are associated with the expression of key immune-related genes. We found that CD8+ T cells are present in sarcoma subtypes and that *PD-L2* is highly expressed. These findings further define potential mechanisms behind the immunotherapy response of specific sarcoma subtypes and can be used to develop more optimal treatments in the future.

## 1. Introduction

Soft tissue sarcomas are group of rare diseases of mesenchymal origin accounting for only 1% of all adult malignancies. These malignancies exhibit different histologic subtypes and have varying clinical behavior, making it difficult to develop a standardized approach for treatment. Despite localized treatment, soft tissue sarcomas recur and often become metastatic at intermediate or high grades. Thus, new approaches to target and treat soft tissue sarcomas are needed [1].

Our group previously identified that preferentially expressed antigen in melanoma (PRAME) is overexpressed in synovial sarcomas and multifocal leiomyosarcomas, suggesting that it may serve as a potential immunotherapy target [2]. Similarly, we showed that melanoma-associated antigen 3 (MAGE-A3) is expressed in undifferentiated pleomorphic sarcoma/myxofibrosarcoma and can be incorporated into immunotherapy techniques [3]. Using next-generation sequencing (NGS), we also identified unique aberrations in intimal sarcomas that can be therapeutically targeted [4].

There is now great interest in applying immunotherapy to sarcomas to immuno-profile the different subtypes and immunomonitor prognosis [5]. In fact, a variety of immunotherapy clinical trials have been initiated or completed in sarcoma patients involving checkpoint inhibitors, adoptive cell therapy, and vaccines [6]. Results from the SARC028 trial revealed that the single-agent pembrolizumab (anti-PD-1) antibody exhibits clinical benefit in specific subsets of sarcoma [7,8]. In addition, there are some promising studies showing that combined checkpoint inhibitors yield a positive tumor response in patients with refractory alveolar soft part sarcoma [9]. Recent work has also focused on understanding the immune microenvironment in sarcoma subtypes, as well as identifying targetable immune markers. Such studies have found that genomically complex sarcomas driven by mutations and/or copy number alterations have higher numbers of tumor-infiltrating lymphocytes, which are associated with increased survival in patients [10,11]. In addition, the immune checkpoints LAG-3 and TIM-3 are frequently expressed in most sarcoma subtypes and associated with PD-1, suggesting that these markers could be targeted along with PD-1 [10].

The aim of this study was to determine whether specific genomic alterations and the expression of infiltrating cytotoxic and suppressive cell type markers identified by NGS warrant further consideration of using immunotherapy to treat certain soft tissue sarcoma subtypes. We interrogated a comprehensive and integrated genomic dataset of adult soft tissue sarcomas [12] to find that immunotherapy-responsive subtypes of sarcoma have a higher mutation load compared to subtypes that do not respond to immunotherapy. In addition, an increased mutation load was found to be associated with the expression of key immune-related genes. Key checkpoint and immune-related genes were also present in all subtypes of sarcoma with low levels of copy number alterations, mutations, amplifications, and deletions. Interestingly, *PD-L2* expression levels were found to be high in all sarcoma subtypes, similar to the expression levels observed in diffuse large B cell lymphoma (DLBC). Lastly, the presence or absence of key immune cells was analyzed using a previously published dataset [13] to reveal that CD8+ T cells are present in all sarcoma subtypes. Altogether, these data provide a better understanding of the immune composition of different sarcoma subtypes.

## 2. Results

### 2.1. Mutational Burden Is Indicative of Immunotherapy Response in Soft Tissue Sarcomas

**Figure 1:**
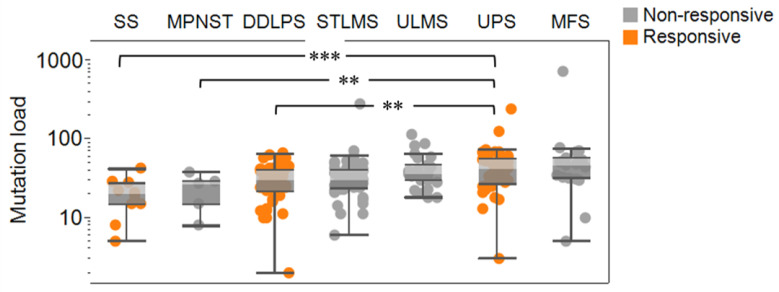
Mutational load (the number of coding mutations) in soft tissue sarcoma subtypes. Subtypes analyzed include synovial sarcoma (SS), malignant peripheral nerve sheath tumor (MPNST), dedifferentiated liposarcoma (DDLPS), soft tissue leiomyosarcoma (STLMS), uterine leiomyosarcoma (ULMS), undifferentiated pleomorphic sarcoma (UPS), and myxofibrosarcoma (MFS). Subtypes are sorted by median mutation load. Orange color indicates subtypes that responded to immunotherapy in SARC028. ** indicates *p* < 0.01, *** indicates *p* < 0.001.

Mutation load plays an important role in characterizing the general immunogenicity of tumors [14]. The mutation load in soft tissue sarcomas tends to be lower than in other tumors [15]. Using TCGA, Figure 1 confirms that mutational burden (the number of coding mutations) is generally low in soft tissue sarcomas. However, sarcomas known to successfully respond to immunotherapy showed a relatively higher load compared to synovial sarcoma (SS), malignant peripheral nerve sheath tumor (MPNST), dedifferentiated liposarcoma (DDLPS), soft tissue leiomyosarcoma (STLMS), uterine leiomyosarcoma (ULMS), undifferentiated pleomorphic sarcoma (UPS), and myxofibrosarcoma (MFS). For example, mutational load was significantly higher in UPS, an immunotherapy-responsive sarcoma subtype, as shown in the multicenter, two-cohort, open-label, phase 2 trial of the immunotherapy pembrolizumab (SARC028) [8], compared to SS (*p* < 0.001), MPNST (*p* < 0.01), and DDLPS (*p* < 0.01); however, one patient with SS and two patients with DDLPS had a tumor response in SARC028. It is important to note that ULMS patients did not show an objective response in SARC028. However, mutational load was not significantly lower in STLMS and ULMS compared to UPS. MFS mutational burden was not significantly different from any other subtype, possibly because of a low sample count and few tumors with either a very low or a very high number of mutations. Furthermore, in SARC028, one SS patient responded to pembrolizumab, but the mutational load in this subtype was shown to be the lowest, suggesting that mutation burden is indicative but not a sufficient predictor of immunotherapy response [16].

### 2.2. Increased Mutation Load Is Associated with the Expression of Immune-Related Genes in Soft Tissue Sarcomas

**Figure 2:**
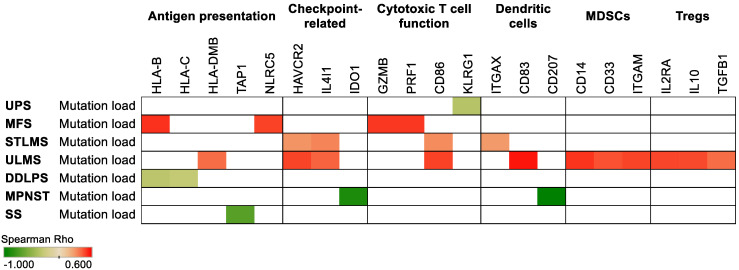
Increased mutation load is associated with the expression of select immune-related genes in soft tissue sarcomas. Red represents positive while green represents negative Spearman Rho correlation coefficients. Only *p* < 0.05 associations are shown. Orange arrows indicate subtypes that responded to immunotherapy in SARC028.

To gain insight into how mutation burden is associated with the immune characteristics of sarcomas, we analyzed the TCGA to determine how mutation burden correlates with the expression of select immune-related genes. Increased mutation load significantly correlated with immune genes involved in antigen presentation, as shown specifically by the Human Leukocyte Antigen (HLA) B and HLA-C genes in DDLPS as well as the Transporter 1 (TAP1) transporter gene in SS (*p* < 0.05) (Figure 2). Furthermore, mutation burden positively correlated with the expression of the HAVCR2 (TIM-3) and IL4I1 checkpoint genes in STLMS and ULMS (*p* < 0.05). Interestingly, higher myeloid-derived suppressor cell (MDSC) and regulatory T cell (Treg) marker gene expression levels were associated (*p* < 0.05) with increased mutation load only in ULMS.

### 2.3. CD8+ T Cells Are Present in All Soft Tissue Sarcoma Subtypes Based on Immune Infiltrate Analyses

**Figure 3:**
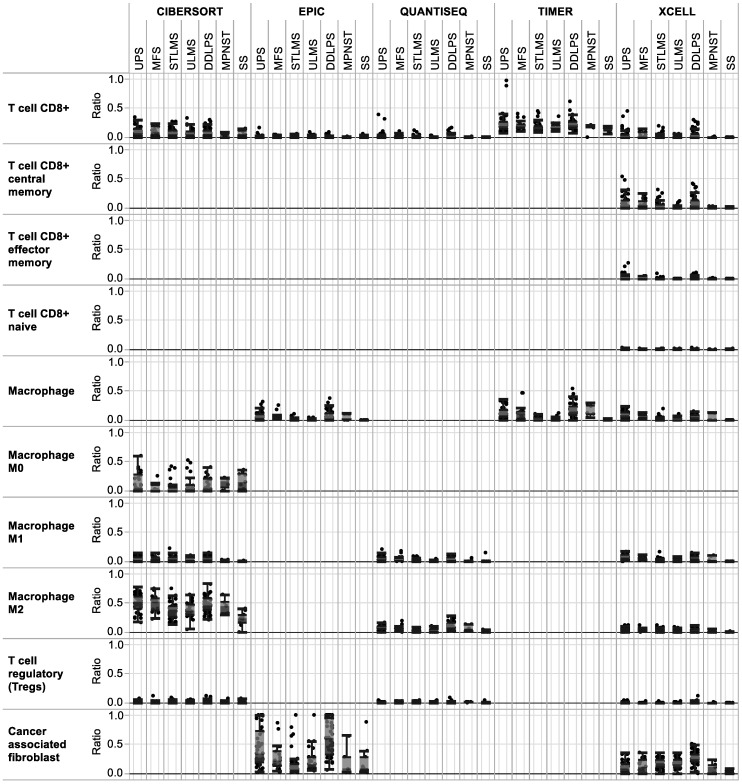
Immune infiltrate analyses using TCGA reveals CD8+ T cell, macrophage, T regulatory cell, and cancer-associated fibroblast levels in all soft tissue sarcoma subtypes. The *y*-axis represents the estimation of the immune infiltration level for a given cell type within the tumor infiltrate.

To understand the composition of the immune infiltrate in the different sarcoma subtypes, multiple algorithms including CIBERSORT, EPIC, QUANTISEQ, TIMER, and XCELL were interrogated to estimate the amount of different immune cell types in the TCGA tumors [13]. As shown in Figure 3, the different algorithms analyzed did not yield similar results, so we limited our analyses to just observing the presence or absence of specific immune cell types. We believe these differences are due to the unique gene sets used by each method. As shown by the different algorithms used to analyze the presence of specific cells, CD8+ T cells were clearly present in all tumor types, indicating that an insufficient response to immunotherapies may not be due to a lack of CD8+ T cells. Others showed that an increase in CD8+ T cells in sarcomas is positively associated with macrophages [17,18]. Based on these results, one possible explanation for a lack of immunotherapy response may be related to suppressive cells and mechanisms, such as M2 macrophages and cancer-associated fibroblasts [19]. Moreover, aneuploidy is associated with decreased levels of immune infiltrates [20,21]. Thus, different genomic karyotypes may also influence immune infiltrates.

### 2.4. Key Immune-Related and Checkpoint GENES Are Expressed in All Sarcoma Subtypes and May Be Appropriate Therapy Targets

**Figure 4:**
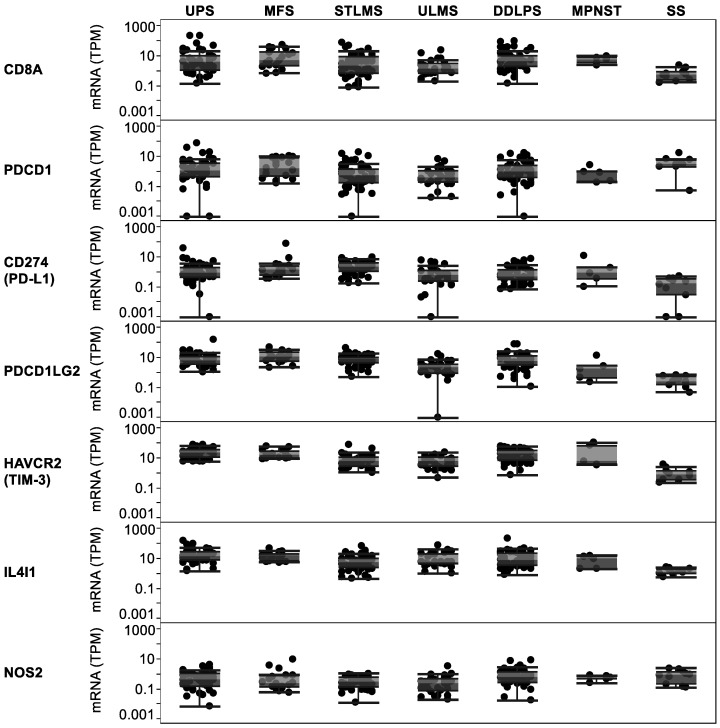
Key immune-related and checkpoint genes are expressed in all soft tissue sarcoma subtypes.

After analyzing the association between immune infiltrates and mutation load in soft tissue sarcomas, we next aimed to specifically evaluate the expression of key genes involved in checkpoint and immunotherapy responses in the different sarcoma subtypes using the TCGA. *CD8A* expression confirmed the presence of CD8A+ T cells in all subtypes (Figure 4). However, the expression of *CD8A* was significantly lower in SS compared with UPS (*p* < 0.05), MFS (*p* < 0.01), STLMS (*p* < 0.001), ULMS (*p* < 0.05), DDLPS (*p* < 0.001), and MPNST (*p* < 0.05). Interestingly, Programmed Cell Death 1 (*PD-1*, *PDCD1*) expression in the SS subtype did not significantly differ from the UPS, MFS, STLMS, and DDLPS subtypes. *PD-L1* (*CD274*) and Programmed Cell Death 1 Ligand 2 (*PD-L2, PDCD1LG2*) expression levels were observable in most samples. The Hepatitis A Virus Cellular Receptor 2 (*HAVCR2, TIM-3*) and Interleukin 4 Induced 1 (*IL4I1)* immune checkpoint-related genes, both of which showed a positive association with mutational burden in STLMS and ULMS, were clearly expressed. In addition, Nitric Oxide Synthase 2 (*iNOS, NOS2*), which was discussed earlier as a target to enhance immunotherapies [22], was also present in all subtypes. The expressed genes listed may be appropriate therapy targets for soft tissue sarcomas.

### 2.5. PD-L2 Expression Levels Are High in Soft Tissue Sarcomas

**Figure 5:**
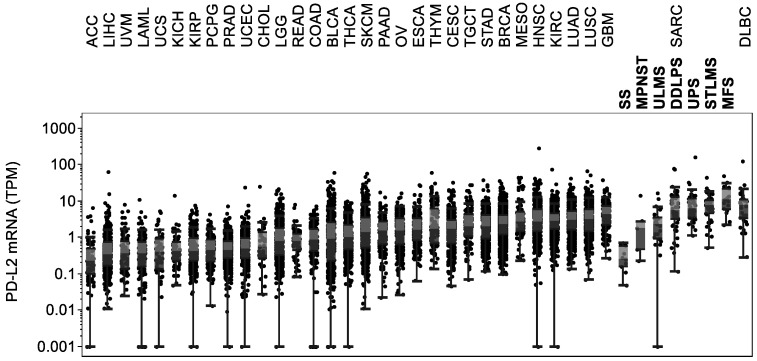
*PD-L2* expression levels are high in soft tissue sarcomas. Tumor types and subtypes are sorted by median *PD-L2* expression levels.

The functions of programmed death-1 receptor ligand 2 (PD-L2) in the immune response are not clear. PD-L2 is a target of interest in cancer and is expressed in sarcomas [23]. Sarcoma tissues showed the second highest expression of PD-L2 compared to all other cancer types based on our TCGA analysis (Figure 5). No significant differences in PD-L2 expression levels were found when comparing sarcoma subtypes to the lymphoid neoplasm DLBC that highly expressed PD-L2.

### 2.6. Immune Checkpoint-Related Gene Copy Number Alterations and Mutations in Soft Tissue Sarcomas

**Figure 6:**
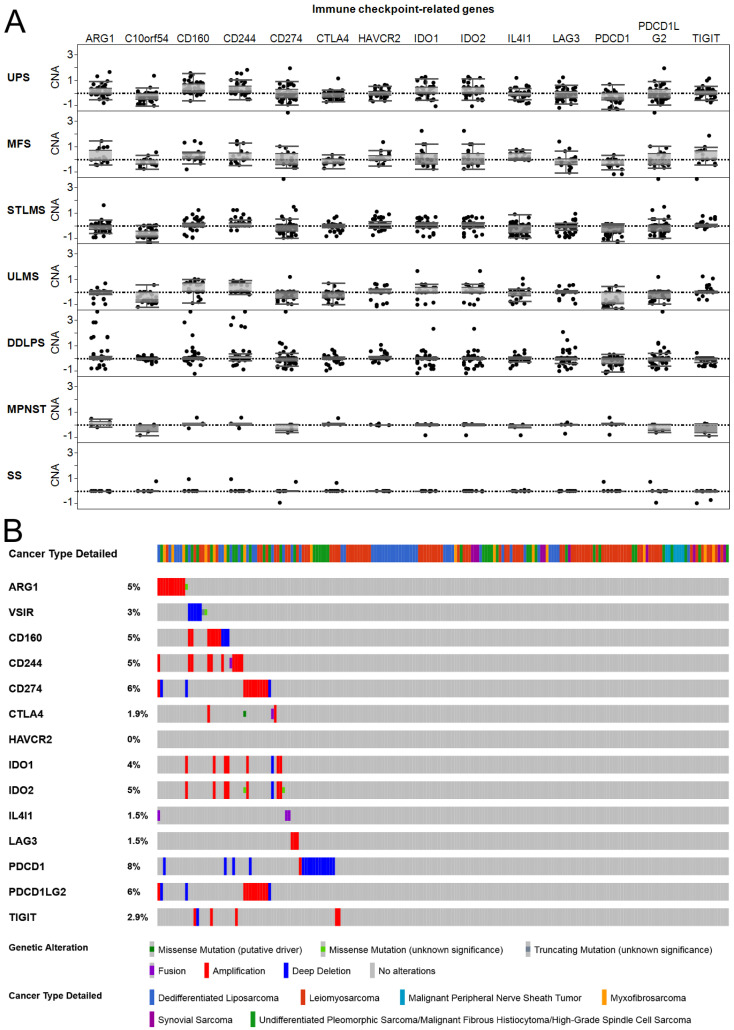
There are low rates of copy number alterations (CNAs) (**A**) and mutations (**B**) of immune checkpoint-related genes in soft tissue sarcomas.

In addition to analyzing the expression of key immune checkpoint genes, copy number alterations, as well as mutations, were analyzed using the TCGA to determine whether alterations were present in sarcoma subtypes. Copy number gains were observed for checkpoint genes (Figure 6A), including the metabolism-associated enzyme arginase 1 (ARG1), in MFS. ARG1 is expressed in immunosuppressive myeloid cells and depletes L-arginine, which is required for T cell proliferation [24]. However, copy number change clustered close to zero, showing very few high gains or deep deletions. Next, cBio portal analysis [25] of immune checkpoint-related genes was performed to show a limited number of mutations, amplifications, or deep deletions (Figure 6B). However, *ARG1* (5% alterations overall), *PD-L1/CD274* (6%), and *PD-L2/PDCD1LG2* (6%) should be further investigated since these genes showed higher mutation, amplification, and deletion rates. As shown by prior work, PD-L1 copy number gains are a predictor for immune checkpoint inhibitor response in sarcoma [26]. Such alterations contribute to tumor mutational load. In general, tumor mutational burden is a predictive biomarker of the immunotherapy response but can vary based on tumor histology [27]. Identifying alterations and mutations in a tumor helps identify response and predicts prognosis.

## 3. Discussion

Soft tissue sarcomas are a group of rare diseases of mesenchymal origin, and they are clinically challenging due to their complex molecular profiling, characterized by over 100 distinct histological subtypes [28]. Surgery, chemotherapy, irradiation, targeted therapy, or immunotherapy are common multidisciplinary approaches used to treat soft tissue sarcomas [29]. However, a deeper understanding of biomarkers and the distinct subtypes is needed to fully combat tumor development, recurrence, and metastasis. Since immunotherapy successfully treats various cancers, there has been interest in its use to treat sarcomas [6].

Immunotherapy does not successfully treat all sarcomas since there are many molecular subtypes that require different therapies [28]. Specifically, understanding the immune composition and microenvironment is critical. The aim of this study was to determine whether the presence of genomic alterations and expression of key immune genes varied across the different sarcoma subtypes for a better understanding of how each distinct type responds to treatment.

We found that sarcomas already known to respond to immunotherapy exhibited a higher mutation load. We concluded that mutation burden is only indicative and not adequate for predicting the success of the immunotherapy response. However, increased mutation load significantly correlated with the expression of immune genes involved in antigen presentation and positively correlated with known checkpoint genes. CD8+ T cells are present in all tumor types to some extent, suggesting that one explanation for a lack of immunotherapy response may be through macrophages and cancer-associated fibroblasts. Another explanation could be that there may be a required threshold in the amount of CD8+ T cells for a specific subtype to respond. Others have shown that despite the presence of CD8+ T cells, different subtypes have different numbers and phenotypical signs of antitumor responsiveness [30]. Similarly, key immune-related and checkpoint genes, such as PD-L2, are expressed in all sarcoma subtypes but have few gene copy number alterations and mutations. A recent study has shown that circulating PD-L2 levels are associated with improved progression-free survival outcomes and are a promising predictor of improvement in the clinical outcome of PD-1 therapy in soft tissue sarcomas [31]. The expression of these key genes suggests opportunities for targeted therapies and prognostic markers.

Taken together, this study advances prior work by providing insight into the gene expression and alteration status of key immune genes in the different soft tissue sarcoma subtypes. These findings contribute to the further understanding of the immune composition behind each subtype and provide insight into treatment strategies. Additional studies need to be performed to confirm the expression of these key immune-related genes at the protein level in human tissue samples for each sarcoma subtype. New combinational strategies may also be developed based on these findings. Furthermore, additional sequencing data will be needed for SS and MPNST since conclusions were based on small sample sizes (*n* = 10 and *n* = 5, respectively). Another limitation to our study is the lack of functional and expressional analyses confirming these findings. Such experiments will be included in future work.

## 4. Materials and Methods

### 4.1. TCGA data

Sarcoma Cancer Genome Atlas (TCGA) mutation, mRNA expression, copy number, and clinical data were used to analyze mutation load, cytotoxic and suppressive cell activity, and the relevance of checkpoint-related genes in DDLPS (*n* = 50), STLMS (*n* = 53), ULMS (*n* = 27), UPS (*n* = 44), MFS (*n* = 17), SS (*n* = 10), and MPNST (*n* = 5). Datasets were provided by previously published studies, including all TCGA exome, RNA sequencing, and clinical data [12], as well as immune infiltrate predictions [13]. All samples were extracted from treatment-naïve patients.

### 4.2. Data Analysis and Visualization

A two-tailed Student t-test was performed to compare two sample groups. Differences were considered statistically significant when *p* < 0.05. cBio portal analyses were performed using https://www.cbioportal.org (accessed on 4 December 2019) [25]. Charts were created using Tableau Desktop software.

## 5. Conclusions and Future Directions

Genomic alterations in sarcoma subtypes may be exploited to develop future immunotherapies. Mutation burden in sarcoma is generally low; however, it may play a role in immunogenicity and in predicting response to immunotherapies. Immune checkpoint-related targets, in addition to PD-1 and PD-L1, warrant further in vitro and in vivo validation and investigation, especially PD-L2, which shows elevated expression compared to several other tumor types. We would like to emphasize that our conclusions are based on a relatively small number of samples for a few subtypes of soft tissue sarcomas, and larger studies will be needed to confirm our results. The most interesting findings are summarized in Table 1.

## Figures and Tables

**Table 1:** Summary of karyotype, mutational load, immune infiltrate, and immune-related marker findings for the different sarcoma subtypes.

STS Subtype	Karyotype	Mutation Load	Immune Infiltrate	Immune-Related Markers
UPS	complex	high	CD8+ T cells; macrophages	PD-L2; PD-L1
MFS	complex	high	CD8+ T cells; macrophages	PD-L2; PD-L1
STLMS	complex	high	CD8+ T cells; macrophages	PD-L2; PD-L1; TIM-3; IL4I1
ULMS	complex	high	CD8+ T cells; macrophages	PD-L2; PD-L1; TIM-3; IL4I1
DDLPS	complex	high	CD8+ T cells; macrophages	PD-L2; PD-L1
MPNST	complex	low	CD8+ T cells; macrophages	PD-L1
SS	simple	low	CD8+ T cells; macrophages	

## Data Availability

All data are available from public TCGA repositories and publications referenced in the paper.
